# Gender Differences in Fears Related to Low-Risk Papillary Thyroid Cancer and Its Treatment

**DOI:** 10.1001/jamaoto.2023.1642

**Published:** 2023-07-06

**Authors:** Anna M. Sawka, Sangeet Ghai, Lorne Rotstein, Jonathan C. Irish, Jesse D. Pasternak, Patrick J. Gullane, Eric Monteiro, Afshan Zahedi, Everton Gooden, Antoine Eskander, Janet Chung, Karen Devon, Jie Su, Wei Xu, Jennifer M. Jones, Amiram Gafni, Nancy N. Baxter, David P. Goldstein

**Affiliations:** 1 Division of Endocrinology, University Health Network and University of Toronto, Toronto, Ontario, Canada; 2 Joint Department of Medical Imaging, University Health Network–Mount Sinai Hospital–Women’s College Hospital, University of Toronto, Toronto, Ontario, Canada; 3 Department of Surgery, University Health Network and University of Toronto, Toronto, Ontario, Canada; 4 Department of Otolaryngology–Head and Neck Surgery/Surgical Oncology, Princess Margaret Cancer Centre, University Health Network and University of Toronto, Toronto, Ontario, Canada; 5 Department of Otolaryngology and Head and Neck Surgery, Mount Sinai Hospital and University of Toronto, Toronto, Ontario, Canada; 6 Division of Endocrinology, Women’s College Hospital and University of Toronto, Toronto, Ontario, Canada; 7 Department of Otolaryngology and Head and Neck Surgery, North York General Hospital and University of Toronto, Toronto, Ontario, Canada; 8 Department of Otolaryngology and Head and Neck Surgery, Sunnybrook Health Sciences Centre and University of Toronto, Toronto, Ontario, Canada; 9 Department of Otolaryngology and Head and Neck Surgery, Trillium Health Partners and University of Toronto, Toronto, Ontario, Canada; 10 Division of Endocrinology, Women’s College Hospital and University of Toronto, Toronto, Ontario, Canada; 11 Department of Biostatistics, Princess Margaret Cancer Centre, University Health Network, Toronto, Ontario, Canada; 12 Department of Biostatistics, Dalla Lana School of Public Health, University of Toronto, Toronto, Ontario, Canada; 13 Department of Psychosocial Oncology, University Health Network and University of Toronto, Toronto, Ontario, Canada; 14 Centre for Health Economics and Policy Analysis, Department of Health Research Methods, Evidence, and Impact, McMaster University, Hamilton, Ontario, Canada; 15 Melbourne School of Population and Global Health, University of Melbourne, Melbourne, Victoria, Australia

## Abstract

**Importance:**

Fear is commonly experienced by individuals newly diagnosed with papillary thyroid cancer (PTC).

**Objective:**

To explore the association between gender and fears of low-risk PTC disease progression, as well as its potential surgical treatment.

**Design, Setting, and Participants:**

This single-center prospective cohort study was conducted at a tertiary care referral hospital in Toronto, Canada, and enrolled patients with untreated small low risk PTC (<2 cm in maximal diameter) that was confined to the thyroid. All patients had a surgical consultation. Study participants were enrolled between May 2016 and February 2021. Data analysis was performed from December 16, 2022, to May 8, 2023.

**Exposures:**

Gender was self-reported by patients with low-risk PTC who were offered the choice of thyroidectomy or active surveillance. Baseline data were collected prior to the patient deciding on disease management.

**Main Outcomes and Measures:**

Baseline patient questionnaires included the Fear of Progression–Short Form and Surgical Fear (referring to thyroidectomy) questionnaires. The fears of women and men were compared after adjustment for age. Decision-related variables, including Decision Self-Efficacy, and the ultimate treatment decisions were also compared between genders.

**Results:**

The study included 153 women (mean [SD] age, 50.7 [15.0] years) and 47 men (mean [SD] age, 56.3 [13.8] years). There were no significant differences in primary tumor size, marital status, education, parental status, or employment status between the women and men. After adjustment for age, there was no significant difference observed in the level of fear of disease progression between men and women. However, women reported greater surgical fear compared with men. There was no meaningful difference observed between women and men with respect to decision self-efficacy or the ultimate treatment choice.

**Conclusions and Relevance:**

In this cohort study of patients with low-risk PTC, women reported a higher level of surgical fear but not fear of the disease compared with men (after adjustment for age). Women and men were similarly confident and satisfied with their disease management choice. Furthermore, the decisions of women and men were generally not significantly different. The context of gender may contribute to the emotional experience of being diagnosed with thyroid cancer and its treatment perception.

## Key Points

**Question:**

Do women and men diagnosed with low-risk papillary thyroid cancer (PTC) experience different fears?

**Findings:**

This cohort study of 153 women and 47 men analyzed baseline questionnaire data in which patients with low-risk PTC were offered the choice of active surveillance or surgery. After adjustment for age, no meaningful difference was observed in the level of fear of disease progression between men and women, but women reported greater surgical fear compared with men.

**Meaning:**

Gender-specific concerns may need to be considered in meeting the educational and support needs of patients with PTC.

## Teaser

This cohort study explores the association between gender and fears of low-risk papillary thyroid cancer disease progression, as well as its potential surgical treatment.

## Introduction

Individuals diagnosed with thyroid cancer are known to experience distressing emotions, including shock, fear, and anxiety.^1,2,3^ Concerns expressed by patients with newly diagnosed low-risk papillary thyroid cancer (PTC) relate to the cancer and its associated future outcome uncertainty as well as the potential risks and consequences of its treatment (eg, thyroidectomy).^4^ It is important to fully understand the fears experienced by individuals newly diagnosed with thyroid cancer to inform supportive care strategies and patient counseling. This understanding may be more fully informed in considering the context of gender.

Gender is reported to be an important factor influencing distress in individuals diagnosed with or surviving cancer.^5,6,7,8,9^ In this secondary analysis from a prospective Canadian cohort study, we compared the initial fears related to thyroid cancer and its treatment between women and men. We specifically explored whether gender was independently associated with fears of the disease and its potential surgical treatment, after adjustment for age. Treatment decisions and confidence in treatment decision-making were also compared between women and men.

## Methods

### Study Design and Participants

We conducted a Canadian single-center, prospective, cohort study in which patients with small, low-risk, untreated PTC (PTC that is <2 cm in maximal diameter and confined to the thyroid) were offered the options of active surveillance or surgery. Study participants were enrolled between May 2016 and February 2021. All patients had a formal surgical consultation prior to enrollment. The study was approved by the University Health Network Research Ethics Board (15-8942), and the details have been previously reported (NCT03271892).^10,11,12^ All participants provided written informed consent for study participation; however, during the pandemic, as per a COVID-19–related study protocol amendment approved by the research ethics board, verbal consent with subsequent written patient co-signature of the consent form was permitted. This study followed the Strengthening the Reporting of Observational Studies in Epidemiology (STROBE) reporting guideline.

### Questionnaires

After enrollment but prior to deciding on PTC management, patients completed baseline written questionnaires. All questionnaires were completed in entirety by all of the participants. These data were supplemented by medical record review. Gender, which was inferred for binary options for sex, specifically “female” and “male”; sociodemographic characteristics; and medical history were self-reported. Baseline questionnaires included the Fear of Progression (of disease)–Short Form^13,14^ and the Surgical Fear questionnaire (instructing the patient to consider the questions in the context of thyroidectomy).^15^ Because the Surgical Fear questionnaire does not contain questions specific to thyroidectomy, we inquired about thyroidectomy-specific fears in 4 similarly formatted additional questions (on potential voice change, requirement for thyroid hormone, hypocalcemia, and scar appearance). For established questionnaires, the scores were estimated as per the instructions of the developers, including respective total scores for Fear of Progression and Surgical Fear questionnaires. The total score of the Fear of Disease Progression questionnaire was estimated on a scale of 5 to 60 (where 60 is the greatest fear). The total score of the Surgical Fear questionnaire was estimated on a scale of 0 to 80 (80 being the greatest fear). The 4 questions on thyroidectomy-specific fears were scored in a manner similar to the Surgical Fear questionnaire (on a scale of 0 to 10 for each question, where 10 is the most afraid), and the results were reported separately from the other questionnaires.

At baseline, we also evaluated participants’ confidence in decision-making about the choice of active surveillance or surgery. We used the Decision Self-efficacy Scale,^16,17^ which was scored according to the developers’ instructions (each question on a scale of 0 [not confident at all] to 4 [very confident], with transformation of the total score out of 100 [where a higher score reflects more confidence]). The final disease management choice of active surveillance or surgery was subsequently reported by patients and noted in the study record.

### Statistical Analysis

We summarized descriptive data according to gender as a binary variable (woman/man), reporting frequency and percentage for categorical data and mean (SD) for continuous data. The difference between women and men was estimated by subtracting the continuous measurement values and the percentages for categorical measurements, respectively, for women minus men. The 95% CIs were estimated for the differences. Cohen *d* values and the 95% CIs were estimated for differences of continuous measurements. Cohen *d* values (standardized mean differences) for effect size estimates were categorized as follows: small effect = 0.2, moderate = 0.5, and large = 0.8 or greater. We constructed multivariable linear regression models incorporating the variables of age and gender for association with total scores for the Fear of Progression–Short Form questionnaire and the Surgical Fear questionnaire. Two statisticians (J.S., W.X.) performed all of the statistical analyses using SAS, version 9.4 (SAS Institute) and R, version 4.1.0 (R Foundation for Statistical Computing).

## Results

### Description of Study Participants

We included 153 women and 47 men in this study. The details of the participant flow have been reported elsewhere.^18^ Women participants were significantly younger (mean [SD] age, 50.7 [15.0] years) than men (mean age difference, 5.6 years; Cohen *d* = 0.38; 95% CI, 0.05-0.71) (Table 1). There were no significant differences in primary tumor size, marital status, education, parental status, or employment status between the women and men (Table 1). Overall, 78% (155 of 200) of study participants chose to undergo active surveillance over surgery. Active surveillance was the choice of 78% of women (120 of 153) and 74% of men (35 of 47) (difference, 4%; 95% CI, −12% to 20%) (Table 1).

**Table 1.** Demographic and Clinical Characteristics of Study Participants According to Gender

Variable	No. (%)		Differences between women and men (95% CI); Cohen *d* effect size estimate for continuous measurements^a^
	Women (n = 153)	Men (n = 47)	
Age, mean (SD), y	50.7 (15.0)	56.3 (13.8)	−5.62 (−10.30 to −0.95); Cohen *d* *=* −0.38 (−0.71 to −0.05)
Marital status			
Single	23 (15)	6 (13)	2 (−29 to 33)
Married	109 (71)	38 (81)	−10 (−25 to 5)
Divorced, separated, or widowed	21 (14)	3 (6)	8 (−23 to 39)
Children			
No	34 (22)	8 (17)	5 (−25 to 35)
Yes	119 (78)	39 (83)	−5 (−19 to 9)
Responsible for children <18 y			
No	97 (63)	27 (57)	6 (−15 to 27)
Yes	56 (37)	20 (43)	−6 (−31 to 19)
Education			
Did not finish high school	15 (10)	2 (4)	6 (−25 to 37)
High school degree	23 (15)	10 (21)	−6 (−35 to 23)
College or university	87 (57)	24 (51)	6 (−17 to 29)
Postgraduate degree	28 (18)	11 (23)	−5 (−34 to 24)
Employment status			
Employed	98 (64)	31 (66)	−2 (−21 to 17)
Student	6 (4)	0	4 (NA)
Full-time homemaker/caregiver	13 (8)	0	8 (NA)
Unemployed	5 (3)	1 (2)	1 (−30 to 32)
Retired	29 (19)	15 (32)	−13 (−41 to 15)
Not working due to disability	2 (1)	0	1 (NA)
Tumor size, mean (SD), mm	11.2 (3.8)	11.8 (3.8)	−0.64 (−1.89 to 0.61); Cohen *d* = −0.17 (−0.50 to 0.16)
Active surveillance chosen over surgery	120 (78)	35 (74)	4 (−12 to 20)
Who took responsibility for the treatment choice			
Patient alone	136 (89)	36 (77)	12 (−3 to 27)
Patient and physician (shared)	17 (11)	11 (23)	−12 (−41 to 17)
Physician alone	0	0	0 (NA)
Someone else (other than patient or physician)	0	0	0 (NA)

Abbreviation: NA, not applicable.

^a^ The difference between women and men was estimated by subtracting the continuous measurement values and the percentages for categorical measurements, respectively, for women minus men. The 95% CIs are shown for the differences. Cohen *d* and the 95% CIs are shown for continuous measurements.

### Gender Comparison of Fear of PTC Disease Progression

The detailed results of the Fear of Progression–Short Form questionnaire are shown in Table 2. In a univariate comparison, the mean total Fear of Progression questionnaire score was 26.2 for women and 23.0 for men (difference, 3.2; Cohen *d* = 0.34; 95% CI, 0.01-0.68). Women more strongly endorsed experiencing physical symptoms such as rapid heartbeat, stomachache, or agitation when anxious compared with men (difference, 0.5; Cohen *d* = 0.39; 95% CI, 0.05-0.72). Women also reported greater fears than men regarding “severe medical treatments” during the course of illness (Cohen *d* = 0.66) and not being able to work due to the illness (Cohen *d* = 0.31).

**Table 2.** Description of the Fear of Disease Progression–Short Form Questionnaire Scores According to Gender

Fears in the context of thyroid cancer (higher score indicates greater fear)	Score, mean (SD)			Difference between women and men; Cohen *d* effect size estimate (95% CI)^a^
	Entire population	Women (n = 153)	Men (n = 47)	
1. Disease may progress	2.6 (1.2)	2.7 (1.2)	2.5 (1.4)	0.2; Cohen *d* = 0.12 (−0.21 to 0.45)
2. Physician’s appointments or periodic examinations	2.2 (1.3)	2.3 (1.3)	2.0 (1.3)	0.3; Cohen *d* = 0.25 (−0.08 to 0.58)
3. Pain	2.2 (1.3)	2.3 (1.2)	2.2 (1.4)	0.1; Cohen *d* = 0.08 (−0.25 to 0.41)
4. Impediment in reaching professional goals because of illness	1.6 (1.0)	1.6 (1.0)	1.6 (1.0)	0; Cohen *d* = 0.02 (−0.31 to 0.35)
5. Endorsement of experiencing physical symptoms such as rapid heartbeat, stomachache, or agitation when anxious	2.0 (1.3)	2.2 (1.3)	1.7 (1.2)	0.5; Cohen *d* = 0.39 (0.05 to 0.72)
6. The possibility of my children contracting the disease	1.9 (1.3)	1.9 (1.3)	1.8 (1.3)	0.1; Cohen *d* = 0.06 (−0.27 to 0.39)
7. The possibility of relying on strangers for activities of daily living	1.5 (1.1)	1.6 (1.1)	1.5 (1.0)	0.1; Cohen *d* = 0.08 (−0.25 to 0.41)
8. Worry about no longer being able to pursue hobbies because of illness	1.6 (1.1)	1.7 (1.1)	1.5 (0.9)	0.2; Cohen *d* = 0.16 (−0.17 to 0.49)
9. Severe medical treatments during the course of my illness	2.2 (1.3)	2.4 (1.3)	1.6 (1.1)	0.8; Cohen *d* = 0.66 (0.33 to 1)
10. Treatment could damage my body	2.5 (1.4)	2.6 (1.4)	2.3 (1.3)	0.3; Cohen *d* = 0.23 (−0.1 to 0.56)
11. Worry about what will become of my family if something should happen to me	2.9 (1.5)	2.9 (1.4)	2.7 (1.5)	0.3; Cohen *d* = 0.19 (−0.14 to 0.52)
12. The thought of not being able to work due to my illness disturbing me	2.0 (1.3)	2.1 (1.3)	1.7 (1.3)	0.4; Cohen *d* = 0.31 (−0.02 to 0.64)
Total score	25.5 (9.6)	26.2 (9.7)	23.0 (9.2)	3.3; Cohen *d* = 0.34 (0.01 to 0.68)

^a^ The difference between women and men was estimated by subtracting the continuous measurement values and the percentages for categorical measurements, respectively, for women minus men. The 95% CIs are shown for the differences. Cohen *d* and the 95% CIs are shown for continuous measurements.

### Gender Comparison of the Surgical Fear and Thyroidectomy-Specific Fears

In general, women reported greater fear of surgery and its potential negative consequences compared with men (Table 3). The total score on the surgical fear questionnaire was significantly higher (ie, worse fear) in women (mean [SD], 34.1 [20.3]) compared with men (mean [SD], 22.0 [17.9]) (difference, 12.1; Cohen *d* = 0.61; 95% CI, 0.28-0.95). Furthermore, women reported more fear than men in response to questions on fears about surgery, anesthesia, postoperative adverse effects (eg, nausea), pain, deteriorating health due to the operation, and the postoperative recovery/potential need for rehabilitation (Table 3, Cohen *d* ranging from 0.41 to 0.59 for respective questions, ie, generally consistent with moderate effect sizes for respective questions). Women also reported more thyroidectomy-specific fears related to potential postoperative voice changes, hypocalcemia, and scar appearance (Table 3, Cohen *d* ranging from 0.49 to 0.70).

**Table 3.** Surgical Fear Questionnaire Results and Thyroidectomy-Related Fears According to Gender

Fears in the context of thyroidectomy (higher score indicates greater fear)	Score, mean (SD)			Women − men difference; Cohen *d* (95% CI)
	Entire population	Women	Men	
**Surgical Fear questionnaire**				
1. Fear of the operation	4.8 (3.3)	5.2 (3.2)	3.4 (3.3)	1.8; Cohen *d* = 0.57 (0.23 to 0.9)
2. Fear of the anesthesia	3.4 (3.4)	3.8 (3.4)	2.0 (2.9)	1.8; Cohen *d* = 0.54 (0.21 to 0.88)
3. Fear of postoperative pain	4.0 (3.1)	4.3 (3.0)	3.0 (3.0)	1.3; Cohen *d* = 0.41 (0.08 to 0.75)
4. Fear of unpleasant postoperative adverse effects	3.9 (3.1)	4.2 (3.1)	3.0 (2.9)	1.2; Cohen *d* = 0.41 (0.07 to 0.74)
5. Fear of deteriorating health due to the operation	4.4 (3.3)	4.8 (3.3)	2.9 (2.9)	1.9; Cohen *d* = 0.59 (0.26 to 0.92)
6. Fear of failure of the operation	3.4 (3.3)	3.7 (3.4)	2.6 (2.9)	1.1; Cohen *d* = 0.34 (0 to 0.67)
7. Fear of not recovering completely from the operation	3.7 (3.2)	4.1 (3.3)	2.7 (2.6)	1.4; Cohen *d* = 0.44 (0.11 to 0.78)
8. Fear of a long rehabilitation after the operation	3.7 (3.1)	4.1 (3.2)	2.4 (2.4)	1.6; Cohen *d* = 0.54 (0.2 to 0.87)
Total score surgical fear questionnaire	31.3 (20.4)	34.1 (20.3)	22.0 (17.9)	12.1; Cohen *d* = 0.61 (0.28 to 0.95)
**Thyroidectomy-specific fears**				
1.Voice change due to the operation^a^	4.4 (3.4)	4.9 (3.4)	3.0 (2.9)	1.8; Cohen *d* = 0.55 (0.22 to 0.89)
2. Requiring thyroid hormone because of the operation^a^	4.7 (3.7)	5.0 (3.7)	3.8 (3.3)	1.2; Cohen *d* = 0.32 (−0.01 to 0.65)
3. Fear of hypocalcemia due to the operation^a^	3.5 (3.4)	3.9 (3.5)	2.2 (2.8)	1.7; Cohen *d* = 0.49 (0.16 to 0.83)
4. Fear of scar appearance^a^	3.1 (3.4)	3.6 (3.5)	1.3 (2.2)	2.3; Cohen *d* = 0.7 (0.37 to 1.04)

^a^ Thyroidectomy-specific fear, not part of the Surgical Fear Questionnaire.

### Multivariable Linear Regression Models Predictive of Fears

Both age and gender were incorporated in respective linear regression models examining the associations with overall scores for respective fear questionnaires. Fear of disease progression was independently inversely associated with age (regression coefficient, −0.26; 95% CI, −0.34 to −0.17 for each year) but not significantly associated with gender (male gender regression coefficient, −1.85; 95% CI, −4.79 to 1.08). Surgical fear was independently inversely associated with age (regression coefficient, −0.29; 95% CI, −0.47 to −0.10) and gender (male, −10.51; 95% CI, −16.97 to −4.05). Thus, younger age was associated with both fear of disease progression and fear of surgery, but women reported more surgical fear compared with men (after adjustment for age). There were no significant interactions between age and gender for either fear of disease progression or surgical fear.

### Gender Comparison of Decision Self-Efficacy and Responsibility for the Decision to Undergo Active Surveillance or Surgery

We observed no meaningful difference in the degree of confidence of women (Decision Self-Efficacy questionnaire score of 93.5 out of 100, where 100 is the highest) compared with men (93.3 out of 100) in deciding on active surveillance or surgery (mean difference in score, 0.1; Cohen *d* = 0.01; 95% CI, −0.31 to 0.34) (eTable in Supplement 1). Furthermore, the responses of women and men were similar with regard to any questions on the thyroid cancer treatment decision-making processes, such as gathering of information and advice, deciphering the information, making the final decision without pressure from others or time, and ultimately informing the care team of their choice (Cohen *d* for respective questions ranging from −0.13 to 0.18, eTable in Supplement 1). We observed no meaningful difference between men and women with respect to taking responsibility for the ultimate treatment choice (Table 1).

## Discussion

In this Canadian cohort of patients with low-risk PTC, women reported moderately greater fear of surgery compared with men (Cohen *d* = 0.61), and this finding was robust in multivariable analysis, after adjustment for age. In contrast, we observed that there were no meaningful gender differences in age-adjusted fear of disease progression. Although the women in our study were generally younger than the men, other factors such as educational attainment and employment status were relatively similar between the groups. In spite of the observed gender differences in surgical fears, women and men reported similar levels of confidence in medical decision-making and, in general, their treatment choices were not meaningfully different from those of men.

Fear may be defined as an emotion experienced in a conscious, subjective state, in response to some form of perceived harm.^19^ It is a normal adaptive response, which may prompt defensive physiological and behavioral changes,^19^ but it may become maladaptive or persistent, relating to anxiety and stress-related disorders.^20^ Epidemiologic literature consistently suggests that women are at higher risk of anxiety as well as trauma and stress-related disorders^20,21,22^ and may experience more adverse symptomatic expression of their anxiety compared with men.^21,22^

Fear of cancer progression (which includes the related construct of fear of recurrence for the purpose of this discussion) is commonly experienced by patients newly diagnosed with cancer.^23^ Younger age is a well-established risk factor for increased fear of cancer progression.^24,25^ A recent systematic review and meta-analysis suggested that women may be more severely affected by fear of cancer recurrence compared with men, but the meta-analysis was subject to significant heterogeneity.^26^ Insufficient accounting for confounders, such as age or disease severity/prognosis, may contribute to the observed heterogeneity in this literature. Although fear of cancer progression has been examined in patients with thyroid cancer and survivors, to our knowledge, analyses of gender differences in fears experienced at the time of treatment decision-making have not been previously reported.

Surgical fear is multifaceted and includes not only fear of the surgery itself but also anesthesia and potential aftereffects, the recovery process, and the postrecovery functional status. In the present study, women and younger individuals reported a greater level of surgical fear. Although recent literature in the field of thyroid surgery has reported on general anxiety and quality of life before and after surgery, there is a paucity of reported literature on fears specifically related to thyroid surgery/anesthesia and postoperative recovery. In examining surgical literature from other fields, women and younger individuals were reported to experience greater fear of brain tumor surgery.^27^ In a systematic review and meta-analysis, the related concept of preoperative anxiety was also reported to be greater in women than men.^28^ There is relatively little qualitative research on this topic; however, in the 1990s, Karlson et al^29^ conducted a focus group study of patients with moderate to severe osteoarthritis, exploring gender differences in preferences for elective total joint replacement. They reported that women expressed much greater “fear of bad outcomes” of surgery than men, referring to concerns about surgical complications, impaired functional status, and anesthesia within this theme.^29^ Similarly, in a qualitative study of patients diagnosed with bladder cancer, Pozzar and Berry^30^ reported that “family relationships and responsibilities served as motivating factors for selecting a course of treatment” in women, but not men. It is possible that consideration of the implications of surgical complications or surgical recovery in the context of gender-specific personal and societal role expectations may contribute to greater surgical fear in some women. A novel feature of the current study is the gender-specific comparison of surgical fear in patients with low-risk PTC at the time of treatment decision-making, which, to our knowledge, has not been previously reported.

The association between fear and medical decision-making participation is important to consider. Jabbour et al^31^ reported on the association between psychological distress and decision-making in a cross-sectional survey study of 597 patients with head and neck cancer (including thyroid cancer) from 4 Australian institutions. They reported that women preferred to take a more active role in cancer treatment decision-making compared with men, in spite of women reporting more psychological distress.^31^ Furthermore, psychological distress was positively associated with wanting to play a more active role in decision-making.^31^ Jabbour et al postulated that provision of additional educational material, such as decision aids, would be helpful in facilitating patient involvement in decision-making.^31^ In the present study, we did not observe any gender-related differences in medical decision-making variables, but it is important to note that the patients in this cohort were aware that they were enrolled in a decision-making study and had ready access to study physicians, who shared information and answered questions related to the disease management options, in part, potentially mitigating distress due to unmet information needs.

### Strengths and Limitations

This study is subject to important strengths, such as a prospective design, careful participant selection based on clinical criteria, and complete data acquisition for the variables described. An important limitation of the study is that on our baseline questionnaire, we categorized gender as a binary variable and inferred it from self-reported sex; thus, data are lacking across varied gender identities. More inclusive options for reporting gender should be incorporated in future research. Other limitations include relative underrepresentation of men (although this is in keeping with thyroid cancer epidemiology), a limited range of racial and ethnic representation, as well as a lack of data collected on health literacy. Another limitation is that we focused on patients with low-risk PTC, and the findings cannot be generalized to patients with more advanced thyroid cancer or other types of cancer. We also did not standardize nor audio- or video-record surgical consultations, which occurred prior to study enrollment. Furthermore, it is important to acknowledge that these data were collected in a Canadian cultural context, and the results may not be generalizable to different societies.

## Conclusions

In this cohort study of Canadian patients with low-risk PTC who were considering primary disease management options, our main finding is that women reported a higher level of surgical fear but not fear of the disease compared with men (after adjustment for age). We are currently following the study population and have expanded the study across multiple Canadian sites to examine longer-term outcomes, including planned comparisons according to gender (NCT04624477).^32^ More research is needed to examine gender-specific fears related to thyroid cancer and its treatment, with attention to gender-inclusive language in framing gender identity. Furthermore, more studies are needed to test interventions intended to reduce surgical fear/perioperative anxiety in patients with thyroid cancer. Nurse-led educational and counseling interventions may be promising in reducing patients’ surgical fear. In a recently published randomized clinical trial of patients with thyroid cancer undergoing total thyroidectomy in Turkey, Altinbas and Gürsoy^33^ reported significantly reduced general anxiety on the day of surgery and a week later, following a nurse-led, web-based patient educational intervention (compared with usual care). Furthermore, in a recent systematic review and meta-analysis of preoperative nursing interventions administered prior to a variety of surgeries, nurse-led interventions, including an informative nursing interview, were also associated with a significant reduction in perioperative anxiety.^34^ Yet these studies may not be directly generalizable to patients with low-risk PTC contemplating the choice of surgery or active surveillance, given that the patients in the aforementioned studies^33,34^ were already committed to having surgery. Pitt and Saucke^35^ have recently developed question prompt–based decision support tools intended to inform patients with low-risk differentiated thyroid cancer about disease management options, including surgery or active surveillance. Furthermore, Wei et al^36^ have proposed a framework for patient counseling, incorporating key factors informing patient treatment choice for low-risk PTC. Multifaceted interventions, including both medical information relevant to the choice (eg, in the form of a decision aid or decision support tool) and a psychosocial support intervention, may be needed to fully address patients’ individualized information and support needs. Furthermore, this study suggests that gender-specific concerns, such those relating to one’s life role (in the workforce and one’s family/social context), may need to be considered in fully meeting the educational and support needs of patients with PTC who are considering their disease management options.

## Notes

Supplement 1. **eTable.** Decision Self-Efficacy in the Choice of Active Surveillance or Surgery According to Gender

Supplement 2.
Canadian Thyroid Cancer Active Surveillance Study Group (Greater Toronto Area) Nonauthor Collaborators

Supplement 3.
Data Sharing Statement
