# Supplementary material for: Gender Differences in Fears Related to Low-Risk Papillary Thyroid Cancer and Its Treatment
Source: JAMA Otolaryngol Head Neck Surg. 2023 Jul 6;149(9):803–10. doi: 10.1001/jamaoto.2023.1642 (PMC10326729; doi:10.1001/jamaoto.2023.1642)
Supplement: Supplement 1. — eTable. Decision Self-Efficacy in the Choice of Active Surveillance or Surgery According to Gender [file jamaotolaryngolheadnecksurg-e231642-s001.pdf]

# Supplemental Online Content

Sawka AM, Ghai S, Rotstein L, et al; for the Canadian Thyroid Cancer Active Surveillance Study Group (Greater Toronto Area). Gender differences in fears related to low-risk papillary thyroid cancer and its treatment. *JAMA Otolaryngol Head Neck Surg.* Published online July 6, 2023. doi:10.1001/jamaoto.2023.1642

**eTable.** Decision Self-Efficacy in the Choice of Active Surveillance or Surgery According to Gender

This supplemental material has been provided by the authors to give readers additional information about their work.

© 2023 Sawka AM et al. *JAMA Otolaryngology–Head & Neck Surgery.*

**eTable. Decision Self-Efficacy in the Choice of Active Surveillance or Surgery According to Gender**

| Feeling confident* in one's ability to: | Entire Population Mean Score (Standard Deviation, SD) | Women Mean Score (SD) | Men Mean Score (SD) | Women- Men difference, Cohen's d and 95% confidence intervals (CI) |
|---|---|---|---|---|
| **1. Get the facts about the choices** | **3.8 (0.5)** | **3.8 (0.5)** | **3.8 (0.5)** | 0, Cohen's d -0.04 (-0.37 to 0.29) |
| **2. Get the facts about the benefits of the choices** | **3.7 (0.5)** | **3.8 (0.5)** | **3.7 (0.5)** | 0, Cohen's d 0.06 (-0.27 to 0.39) |
| **3. Get the facts about the risks and side effects of the choices** | **3.6 (0.7)** | **3.6 (0.7)** | **3.7 (0.6)** | -0.1, Cohen's d -0.13 (-0.46 to 0.2) |
| **4. Understand the information to make a choice** | **3.7 (0.5)** | **3.7 (0.5)** | **3.7 (0.6)** | 0, Cohen's d -0.02 (-0.35 to 0.31) |
| **5. Ask questions without feeling dumb** | **3.8 (0.5)** | **3.8 (0.5)** | **3.9 (0.4)** | 0, Cohen's d -0.1 (-0.42 to 0.23) |
| **6. Express personal concerns about each choice** | **3.8 (0.5)** | **3.8 (0.5)** | **3.7 (0.5)** | 0.1, Cohen's d 0.18 (-0.15 to 0.51) |

© 2023 Sawka AM et al. *JAMA Otolaryngology–Head & Neck Surgery*.

| | | | | |
|---|---|---|---|---|
| **7. Ask advice** | **3.8 (0.4)** | **3.8 (0.4)** | **3.8 (0.4)** | 0, Cohen's d 0.05 (-0.28 to 0.38) |
| **8. Figure out the choice most suited to oneself** | **3.7 (0.5)** | **3.7 (0.5)** | **3.6 (0.6)** | 0.1, Cohen's d 0.12 (-0.2 to 0.45) |
| **9.Handle unwanted pressure from others about the choice** | **3.5 (0.7)** | **3.5 (0.7)** | **3.5 (0.8)** | 0, Cohen's d 0.01 (-0.31 to 0.34) |
| **10. Inform the clinical team about what is best for me** | **3.8 (0.5)** | **3.8 (0.5)** | **3.8 (0.5)** | -0.1, Cohen's d -0.1 (-0.43 to 0.23) |
| **11.Delay the decision if I need more time** | **3.9 (0.4)** | **3.9 (0.4)** | **3.8 (0.4)** | 0.1, Cohen's d 0.13 (-0.2 to 0.46) |
| **OVERALL DECISION SELF-EFFICACY QUESTIONNAIRE SCORE (Scored out of 100, where 100 is the best)** | **93.4 (9.6)** | **93.5 (9.5)** | **93.3 (10.2)** | 0.1, Cohen's d 0.01 (-0.31 to 0.34) |

*A higher score indicates greater confidence

© 2023 Sawka AM et al. *JAMA Otolaryngology–Head & Neck Surgery*.
