# Supplementary material for: Gender Differences in Fears Related to Low-Risk Papillary Thyroid Cancer and Its Treatment
Source: JAMA Otolaryngol Head Neck Surg. 2023 Jul 6;149(9):803–10. doi: 10.1001/jamaoto.2023.1642 (PMC10326729; doi:10.1001/jamaoto.2023.1642)
Supplement: Supplement 2. — Canadian Thyroid Cancer Active Surveillance Study Group (Greater Toronto Area) Nonauthor Collaborators [file jamaotolaryngolheadnecksurg-e231642-s002.pdf]

Supplemental Online Content: Nonauthor Collaborators
*First name, last name, and suffix (if applicable) are required and will appear in PubMed.

*Group Name(s): Canadian Thyroid Cancer Active Surveillance Study Group (Greater Toronto Area)							
***First Name and Middle Initial(s)**	***Last Name**	***Suffix (eg, Jr, III)**	Academic Degrees	Institution	Location (city, state/province, country)	Role or Contribution, eg, chair, principal investigator	Group (if more than 1 Group listed in the byline) and/or Subgroup (eg, Steering Committee)
Avik	Banerjee		MD	Grand River Hospital	Kitchener, Ontario, Canada	Clinical collaborator	
Vinita	Bindlish		MD	Grand River Hospital	Kitchener, Ontario, Canada	Clinical collaborator	
Maky	Hafidh		MD	Grand River Hospital	Kitchener, Ontario, Canada	Clinical collaborator	
Jose	Prudencio		MD	Grand River Hospital	Kitchener, Ontario, Canada	Clinical collaborator	
Vinod	Bharadwaj		MD	Grand River Hospital	Kitchener, Ontario, Canada	Clinical collaborator	
Denny	Lin		MD	Guelph General Hospital	Guelph, Ontario, Canada	Clinical collaborator	
Laura	Whiteacre		MD	Humber River Hospital	Toronto, Ontario, Canada	Clinical collaborator	
Eric	Arruda		MD	Lakeridge Health	Oshawa, Ontario, Canada	Clinical collaborator	
Artur	Gevorgyan		MD	Lakeridge Health	Oshawa, Ontario, Canada	Clinical collaborator	
Marshall	Hay		MD	Independent Practice	Toronto, Ontario, Canada	Clinical collaborator	
Philip	Solomon		MD	Mackenzie Health Hospital	Richmond Hill, Ontario, Canada	Clinical collaborator	
Ian	Witterick		MD	Mount Sinai Hospital	Toronto, Ontario, Canada	Clinical Collaborator	
Allan	Vescan		MD	Mount Sinai Hospital	Toronto, Ontario, Canada	Clinical Collaborator	
Manish	Shah		MD	North York General Hospital	Toronto, Ontario, Canada	Clinical Collaborator	
Michael	Chang		MD	Scarborough Health Network (Centenary Hospital)	Scarborough, Ontario, Canada	Clinical Collaborator	

Supplemental Online Content: Nonauthor Collaborators
*First name, last name, and suffix (if applicable) are required and will appear in PubMed.

***First Name and Middle Initial(s)**	***Last Name**	***Suffix (eg, Jr, III)**	Academic Degrees	Institution	Location (city, state/province, country)	Role or Contribution, eg, chair, principal investigator	Group (if more than 1 Group listed in the byline) and/or Subgroup (eg, Steering Committee)
Andres	Gantous		MD	St. Joseph's Health Centre	Toronto, Ontario, Canada	Clinical Collaborator	
Jennifer	Anderson		MD	St. Michael's Hospital	Toronto, Ontario, Canada	Clinical Collaborator	
Vinay	Fernandes		MD	Scarborough Health Network (General Hospital)	Scarborough, Ontario, Canada	Clinical Collaborator	
Sumeet	Anand		MD	Scarborough Health Network (Birchmount Hospital, Centenary Hospital, General Hospital)	Scarborough, Ontario, Canada	Clinical Collaborator	
Danny	Enepekides		MD	Sunnybrook Health Sciences Centre	Toronto, Ontario, Canada	Clinical Collaborator	
Kevin	Higgins		MD	Sunnybrook Health Sciences Centre	Toronto, Ontario, Canada	Clinical Collaborator	
Ilana J.	Halerin		MD	Sunnybrook Health Sciences Centre	Toronto, Ontario, Canada	Clinical Collaborator	
Karim	Nazarali		MD	Trillium Health Partners	Toronto, Ontario, Canada	Clinical Collaborator	
Lorne	Segall		MD	Trillium Health Partners	Toronto, Ontario, Canada	Clinical Collaborator	DECEASED (2022)
John	de Almeida		MD	University Health Network	Toronto, Ontario, Canada	Clinical Collaborator	
Ralph	Gilbert		MD	University Health Network	Toronto, Ontario, Canada	Clinical Collaborator	
Shereen	Ezzat		MD	University Health Network	Toronto, Ontario, Canada	Clinical Collaborator	
Richard W.	Tsang		MD	University Health Network	Toronto, Ontario, Canada	Clinical Collaborator	
Mark	Korman		MD	William Osler Health System	Brampton, Ontario, Canada	Clinical Collaborator	
