# Supplementary material for: Gender Differences in Fears Related to Low-Risk Papillary Thyroid Cancer and Its Treatment
Source: JAMA Otolaryngol Head Neck Surg. 2023 Jul 6;149(9):803–10. doi: 10.1001/jamaoto.2023.1642 (PMC10326729; doi:10.1001/jamaoto.2023.1642)
Supplement: Supplement 3. — Data Sharing Statement [file jamaotolaryngolheadnecksurg-e231642-s003.pdf]

Data Sharing Statement

Sawka. Gender Differences in Fears Related to Low-Risk Papillary Thyroid Cancer and Its Treatment. *JAMA Otolaryngol Head Neck Surg*. Published July 06, 2023. doi:10.1001/jamaoto.2023.1642

Data
Data available: No
